# Progranulin deficiency does not exacerbate TDP-43 pathology in TDP-43 transgenic mouse models

**DOI:** 10.1038/s44400-025-00020-4

**Published:** 2025-07-21

**Authors:** Cha Yang, Tuancheng Feng, Fenghua Hu

**Affiliations:** Department of Molecular Biology and Genetics, Weill Institute for Cell and Molecular Biology, Cornell University, Ithaca, NY USA

**Keywords:** Cellular neuroscience, Molecular neuroscience

## Abstract

The progranulin (PGRN) protein is tightly linked with TDP-43 proteinopathy in neurodegenerative diseases. However, how PGRN regulates TDP-43 proteinopathy remains unclear. In this study, we investigated the effect of PGRN loss on TDP-43 pathology in the TDP-43^Q331K^ knock-in mice expressing an ALS-linked TDP-43 mutation at the endogenous level, and in the transgenic mice overexpressing human TDP-43 in neurons. We found that PGRN deficiency leads to mild glial activation and behavioral deficits in TDP-43^Q331K^ mice without inducing typical TDP-43 pathology. RNA-seq analysis reveals upregulation of immune pathways and downregulation of myelination-related pathways in PGRN-deficient TDP-43^Q331K^ mice. In addition, we observed myelination defects in human TDP-43 transgenic mice, but PGRN loss does not exacerbate TDP-43 pathology, myelination defects, and motor deficits in this mouse strain. Our studies demonstrated that PGRN deficiency exacerbates behavioral deficits and glial pathology caused by TDP-43 Q331K mutation but has minimal effect on TDP-43 pathology in mouse models.

## Introduction

Transactivation response (TAR) DNA-binding protein 43 (TDP-43), encoded by the *Tardbp* gene, is a DNA/RNA binding protein involved in multiple RNA-related processes^1,2^. In normal conditions, TDP-43 is predominantly localized in the nucleus but shuttles between the nucleus and cytoplasm to exert various functions, including regulation of RNA splicing, RNA trafficking, and RNA stability^2,3^. Due to its critical functions in RNA metabolism and other cellular processes, TDP-43 is essential for maintaining cellular health. TDP-43 proteinopathy, characterized by the accumulation of the hyper-phosphorylated, fragmented, and aggregated TDP-43 in the cytoplasm and depletion of TDP-43 from the nucleus, is a pathologic hallmark of many neurodegenerative diseases, including amyotrophic lateral sclerosis (ALS)^4,5^, frontotemporal lobar degeneration (FTLD)^5–7^, Alzheimer’s disease (AD)^8–10^, Huntington’s diseases (HD)^11^, and limbic-predominant age-related TDP-43 encephalopathy (LATE)^12,13^. However, molecular pathways leading to TDP-43 proteinopathy are still poorly understood.

Haploinsufficiency of progranulin (PGRN) protein caused by mutations in the granulin (*GRN*) gene is known to be a main cause of FTLD with TDP-43 proteinopathy^14–16^. In addition, the granulin (*GRN*) gene has also been identified as a major risk gene for LATE^17,18^, indicating an important role of PGRN in TDP-43 proteinopathy. However, little is known about how PGRN loss of function leads to TDP-43 proteinopathy. PGRN is an evolutionarily conserved glycoprotein comprised of 7.5 granulin repeats^19–21^, with critical roles in inflammation^22^. At the cellular level, PGRN is either secreted to the extracellular space or sorted into the lysosome^23,24^. Within the lysosome, PGRN is processed into granulin peptides, which are critical for proper lysosomal functions, especially during aging^23,24^. Since lysosomal dysfunction and neuroinflammation have both been linked to TDP-43 proteinopathy^25–27^, lysosomal dysfunction and enhanced neuroinflammation caused by PGRN loss may contribute to TDP-43 proteinopathy.

Despite the strong link between PGRN and TDP-43 proteinopathy in humans, minimal alteration in TDP-43 has been observed in PGRN-deficient mice^28^. To investigate the role of PGRN in TDP-43 proteinopathy, we utilize two TDP-43 mouse models. One is the TDP-43^Q331K^ knock-in mouse line expressing TDP-43 Q331K mutant, which is associated with ALS, at endogenous levels. This mouse line does not develop cytoplasmic aggregation or nuclear loss of TDP-43 but shows slightly increased levels of TDP-43 in the nucleus^29^. Another one is the transgenic mouse line overexpressing the wild-type human TDP-43 in all neurons in the central nervous system, which shows accumulation of nuclear and cytoplasmic TDP-43 aggregates in neurons^30^. In both mouse models, we failed to detect significant effects of PGRN deficiency on TDP-43 protein levels, distribution, and solubility, which suggests that mouse models might not be an ideal system to study the role of PGRN in TDP-43 proteinopathy.

## Results

### PGRN deficiency does not induce TDP-43 pathology in TDP-43^Q331K^ mice

**Fig. 1:**
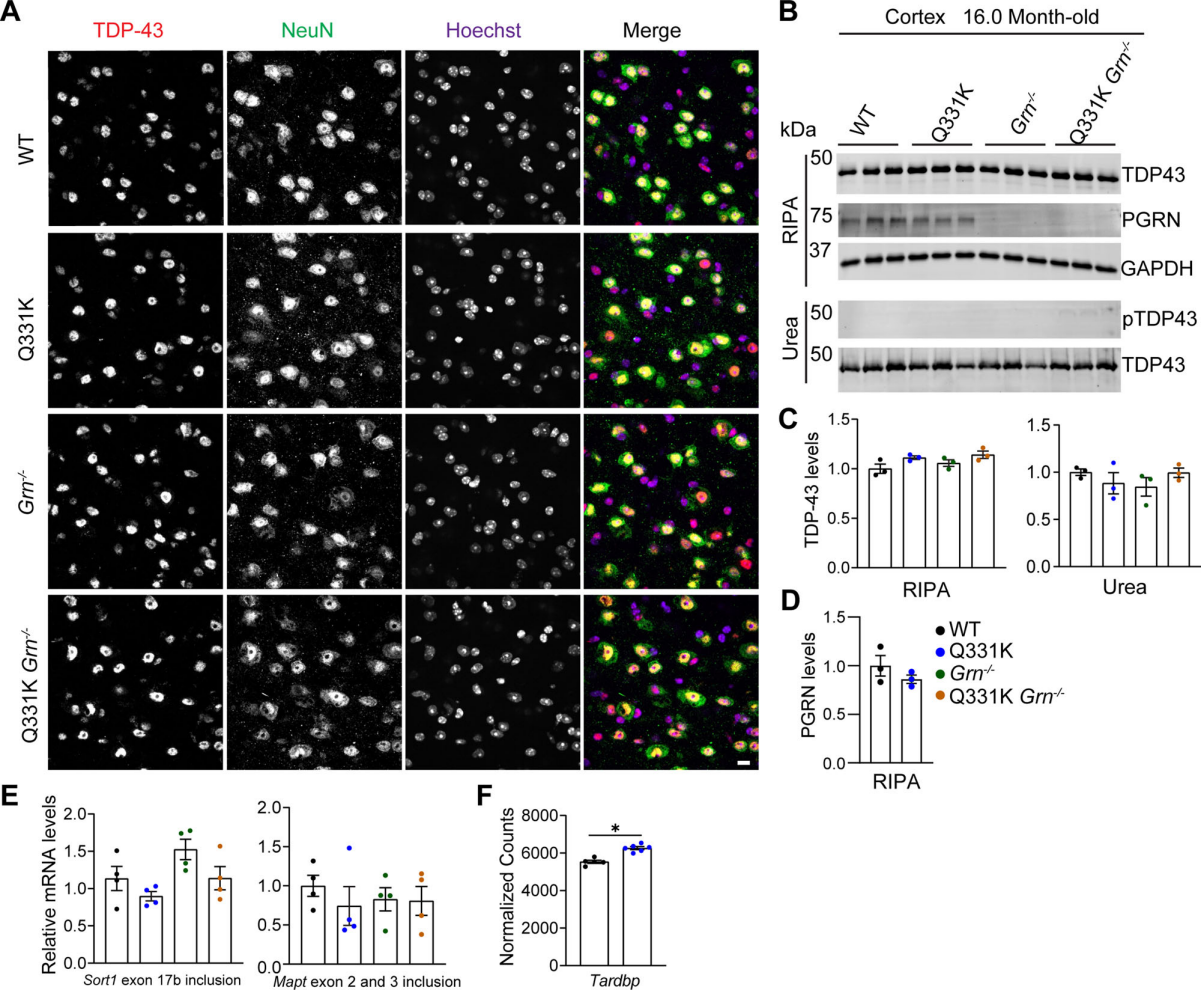
PGRN loss does not exacerbate TDP-43 pathology in TDP-43^Q331K/Q331K^ mice. **A** Immunostaining of TDP-43 and NeuN in brain sections from 16-month-old mice WT, TDP-43^Q331K/Q331K^ (Q331K), *Grn*^*−/−*^, TDP-43^Q331K/Q331K^ *Grn*^*−/−*^ (Q331K *Grn*^*−/−*^) mice. Representative images from the cortex were shown. Scale bar, 10 µm. **B** Analysis of TDP-43, phosphorylated TDP-43 (pS409/410), and PGRN levels in cortical lysates from 16-month-old mice of the indicated genotypes. **C** TDP-43 levels in RIPA- and urea-soluble fractions were quantified. Data are presented as mean ± SEM from 3 mice per group (*n* = 3). One-way ANOVA tests with Bonferroni’s multiple comparisons. **D** PGRN levels in RIPA soluble fractions were quantified and normalized to GAPDH. Data are presented as mean ± SEM from 3 mice per group (*n* = 3). **E** Total RNAs were extracted from the cortex of 10-month-old WT, TDP-43^Q331K/Q331K^, *Grn*^*−/−*^, TDP-43^Q331K/Q331K^*Grn*^*−/−*^ male mice, and the RT-qPCR was performed to analyze the splicing changes in *Sort1* exon 17b (left) and *Mapt* exons 2 and 3 (right). The relative mRNA levels of transcripts including or excluding exons 2 and 3 represent the inclusion of *Mapt* exons 2 and 3. Data are presented as mean ± SEM (*n* = 4 mice per genotype). *p*-values were determined using one-way ANOVA tests with Bonferroni’s multiple comparisons. **F** Expression levels of *Tardbp* in WT and Q331K mice. Total RNAs were extracted from the cortex of 10-month-old WT and Q331K male mice, and the RNA-seq was performed to analyze gene expression changes. Normalized read counts are shown. Data are presented as mean ± SEM (*n* = 5-6 mice per genotype). *p < 0.05, unpaired two-tailed Student's t-test.

Mutations in TDP-43 have been genetically linked to ALS and FTLD^31–33^. Mice expressing the ALS-linked TDP-43 mutant Q331K (TDP-43^Q331K^) at the endogenous TDP-43 loci do not display typical TDP-43 pathology, such as cytoplasmic aggregation and nuclear loss of TDP-43. Instead, they show increased TDP-43 levels due to the perturbed autoregulation caused by the Q331K mutation^29^. To investigate the role of PGRN in TDP-43 pathology, we crossed TDP-43^Q331K^ mice with *Grn*^*−/−*^ mice to assess how PGRN loss affects TDP-43 proteinopathy in this sensitized background. We first determined the effect of PGRN deficiency on TDP-43 homeostasis in the TDP-43^Q331K^ mice. We failed to detect cytoplasmic TDP-43 accumulation in neurons in the cortex region of *Grn*^*−/−*^, TDP-43^Q331K/Q331K^, or TDP-43^Q331K/Q331K^ *Grn*^*−/−*^ mice, even at 16 months of age (Fig. 1A), suggesting PGRN loss does not lead to the cytoplasmic mislocalization of TDP-43 in these mice. Next, we measured the total TDP-43 levels and phosphorylated TDP-43 levels (pS409/410) using a validated pS409/410 antibody (Supplemental Fig. 1) in the 16.0-month-old mouse brain cortex. TDP-43 total levels, solubility, and phosphorylation state are not altered by PGRN loss in TDP-43^Q331K/Q331K^ mice (Fig.1B, C). In addition, TDP-43 Q331K has little effect on PGRN protein levels (Fig.1B, D). Collectively, these results suggest that PGRN loss does not trigger any obvious TDP-43 pathology in the brain of TDP-43^Q331K^ mice.

### PGRN ablation leads to upregulation of immune pathways and downregulation of myelination-related pathways in TDP-43^Q331K^ mice

**Fig. 2:**
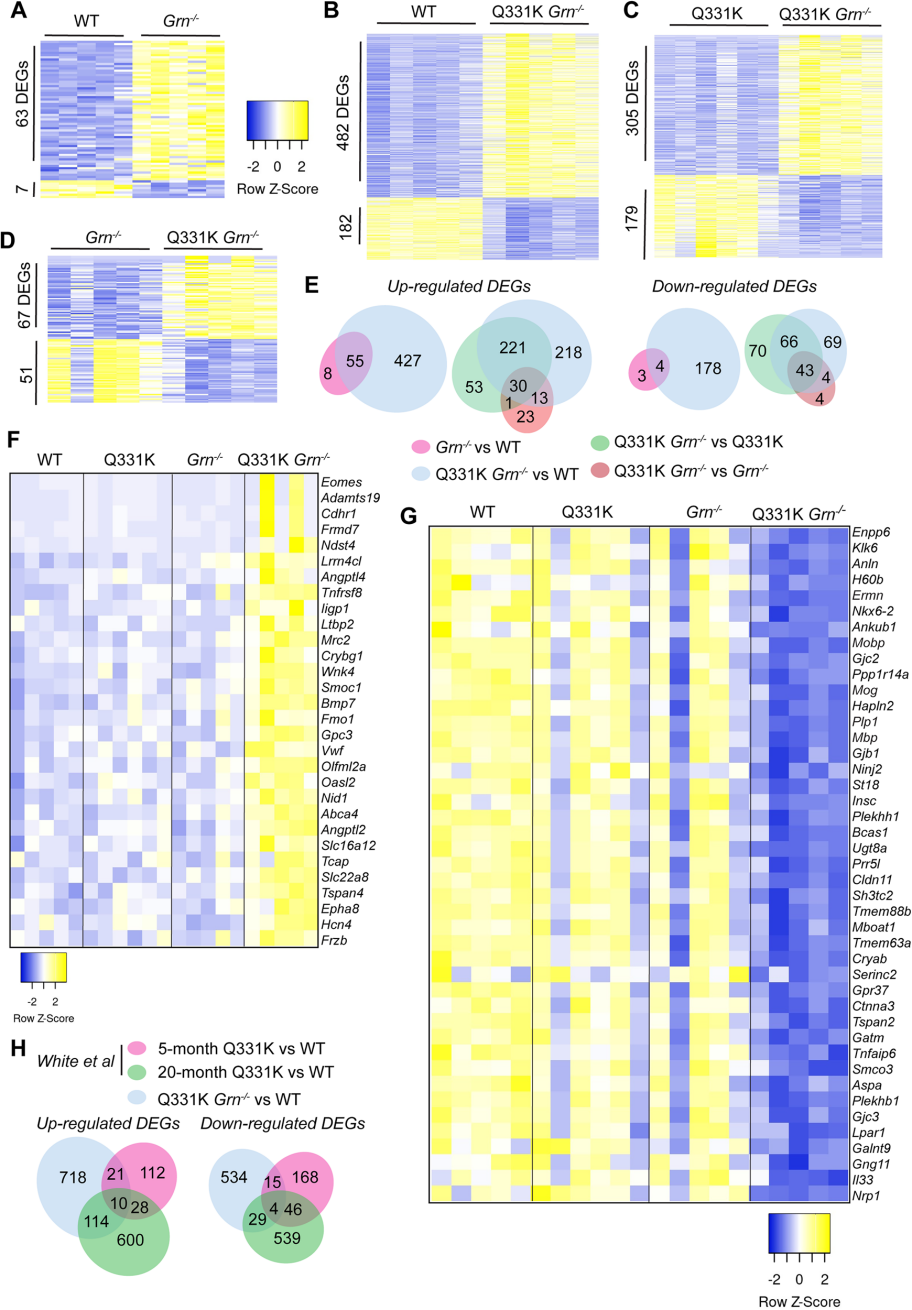
Gene expression changes in the brain of TDP-43^Q331K/Q331K^*Grn*^*−/−*^ mice. Total RNAs were extracted from the cortex of 10-month-old WT, TDP-43^Q331K/Q331K^ (Q331K), *Grn*^*−/−*^, and TDP-43^Q331K/Q331K^*Grn*^*−/−*^ (Q331K *Grn*^*−/−*^) male mice, and the RNA-seq was performed to analyze gene expression changes (*n* = 5–6 mice per genotype). **A** Differentially expressed genes (DEGs) with FDR ≤ 0.05 and absolute log2(FC) ≥ 0.5 between WT and *Grn*^*−/−*^ are plotted using Heatmapper. **B** DEGs identified in the TDP-43^Q331K/Q331K^*Grn*^*−/−*^ compared with WT (FDR ≤ 0.05, absolute log2(FC) ≥ 0.5). **C** Heatmap showing the expression of the DEGs identified in the TDP-43^Q331K/Q331K^*Grn*^*−/−*^ compared with TDP-43^Q331K/Q331K^ (FDR ≤ 0.05, absolute log2(FC) ≥ 0.5). **D** Heatmap showing the expression of the DEGs identified in the TDP-43^Q331K/Q331K^*Grn*^*−/−*^ compared with *Grn*^*−/−*^ (FDR ≤ 0.05, absolute log2(FC) ≥ 0.5). **E** Venn diagrams showing the overlap of the DEGs identified in the indicated comparisons. **F** Heatmap illustrating the expression of the significantly upregulated genes (log2FC ≥ 1) identified in the TDP-43^Q331K/Q331K^*Grn*^*−/−*^ mice compared with WT. **G** Heatmap illustrating the expression of the significantly downregulated genes (log2FC ≤ −1) identified in the TDP-43^Q331K/Q331K^*Grn*^*−/−*^ mice compared with WT. **H** Venn diagrams showing the overlap of the DEGs identified in this study and the previous study by White et al. DEGs (FDR ≤ 0.05) identified in the TDP-43^Q331K/Q331K^*Grn*^*−/−*^ mouse cortical samples were compared to those identified in the 5- and 20-month-old TDP-43^Q331K/Q331K^ mouse cortical samples from the study of White et al.

TDP-43^Q331K^ has been shown to cause alterations in RNA splicing^29^. To determine whether PGRN loss affects TDP-43 function, we performed RT-qPCR to analyze changes in the splicing of known TDP-43 targets and RNA-seq analysis to identify gene expression alterations. A previous study has reported that the TDP-43 Q331K mutation leads to decreased inclusion of *Sort1* exon 17b and increased inclusion of *Mapt* exons 2 and 3^29^. However, we did not observe significant changes in *Sort1* or *Mapt* splicing in RNA samples from the cortex of TDP-43^Q331K/Q331K^, *Grn*^*−/−*^ or TDP-43^Q331K/Q331k^*Grn*^*−/−*^ mice (Fig.1D). In addition, RNA-seq analysis revealed increased *Tardbp* expression in the cortex of 10-month-old TDP-43^Q331K/Q331K^ mice, as demonstrated by increased normalized read counts in TDP-43^Q331K/Q331K^ samples compared to the WT controls (Fig. 1F), confirming the dysregulation of *Tardbp* expression by Q331K mutation in these mice. However, no other significant gene expression changes were detected (Supplementary Data 1). In contrast, significant gene expression changes were identified in *Grn*^*−/−*^ mouse brain, with 63 upregulated and 7 downregulated genes compared to WT controls (Fig. 2A), which are further exacerbated in TDP-43^Q331K/Q331K^ *Grn*^*−/−*^ mouse brain with 482 upregulated and 182 downregulated genes compared to WT (Fig. 2B–E). The expression changes of the DEGs ( | log2FC | ≥1.0) identified in TDP-43^Q331K/Q331K^ *Grn*^*−/−*^ mice versus WT mice were shown in the heatmap (Fig. 2F, G). We further compared the DEGs identified in the TDP-43^Q331K/Q331K^ *Grn*^*−/−*^ mice to those identified in the cortical samples from 5- and 20-month-old TDP-43^Q331K/Q331K^ mice in the previous study^29^. When comparing DEGs with an FDR ≤ 0.05 and FC ≥ 1.5, only five upregulated genes (*Car3*, *Dusp27*, *Lilra5*, *Prg4*, and *Tfap2d*) overlap with those identified in 20-month-old TDP-43^Q331K/Q331k^ mice. However, when using an FDR ≤ 0.05 (without an FC cutoff), more overlap is observed with DEGs from either the 5- or 20-month-old TDP-43^Q331K/Q331k^ mice (Fig. 2H). The discrepancy in the gene expression alterations in the TDP-43^Q331K^ discrepancy in our study versus previous studies^29^ could be attributed to several factors, such as differences in mouse housing environment or technical variations in RNA sequencing and analysis. The key methodology variations include the library preparation method and sequencing depth. Our study used rRNA depletion, which retains both mRNA and non-coding RNAs, potentially requiring deeper sequencing to achieve sufficient coverage of protein-coding genes. In contrast, the previous study employed poly(A) enrichment, which selectively targets mRNA and typically yields cleaner gene expression signals at lower sequencing depths. The prior study sequenced a library of 50 million 100 bp paired-end reads, providing higher read counts for enhanced statistical power in differential expression analysis. Our approach used 40 million 150 bp paired-end reads, which, while offering longer read lengths for alignment accuracy, resulted in fewer total reads. Moreover, differences in analytical pipelines (e.g., alignment tools, DEG-calling algorithms, or significance thresholds) may further contribute to the discrepancy.

**Fig. 3:**
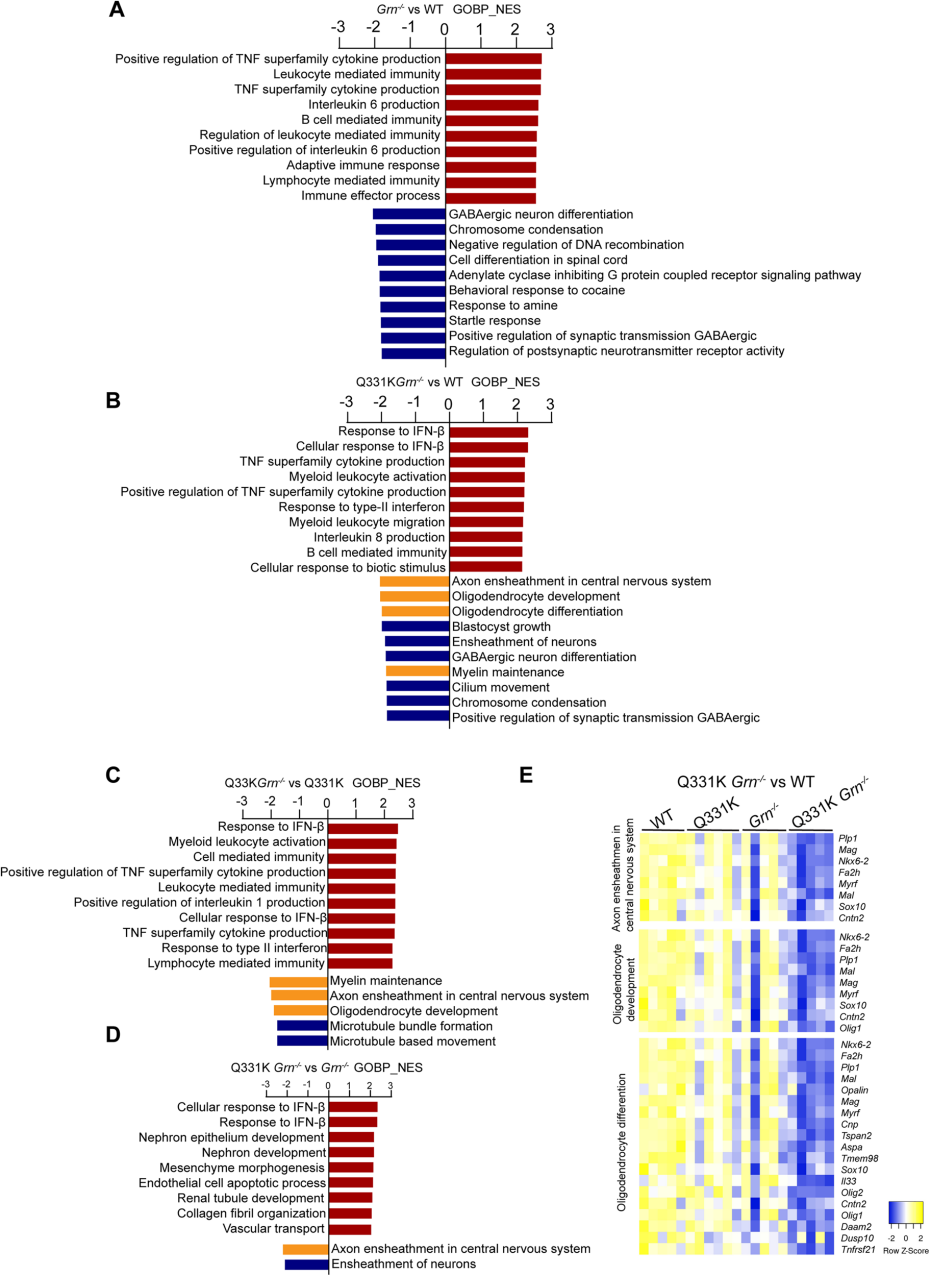
Pathways affected by PGRN deficiency or TDP-43^Q331K^ mutation in mouse brain. **A**–**D** Pathway enrichment analysis was performed by GSEA using the gene sets of gene ontology biological process (GOBP). The significantly upregulated and downregulated gene sets (FDR *q*-value <0.1) identified in the indicated comparisons are listed. The gene sets with normalized enrichment score (NES) ＞ 1.0 are upregulated, and those with NES < −1 are downregulated in the indicated comparisons. Myelination-related pathways were highlighted in orange. **E** Expression changes of the DEGs (FDR ≤ 0.05, absolute log2(FC) ≥ 0.5) identified in axon ensheathment in the central nervous system, oligodendrocyte development, and oligodendrocyte differentiation pathways in TDP-43^Q331K/Q331K^ *Grn*^*−/−*^ brain samples compared to WT based on GSEA results.

Gene set enrichment analysis (GSEA) of all the genes revealed upregulation of immune-related pathways in *Grn*^*−/−*^ mice compared with WT, as well as in TDP-43^Q331K/Q331K^ *Grn*^*−/−*^ mice compared with WT, TDP-43^Q331K/Q331K^, or *Grn*^*−/−*^ (Fig. 3A–D), reflecting immune activation in the cortex in these mice. Notably, several pathways related to myelination, including axon ensheathment in the central nervous system and oligodendrocyte development and differentiation, were significantly downregulated in TDP-43^Q331K/Q331K^ *Grn*^*−/−*^ mice compared to WT or TDP-43^Q331K/Q331K^ (Fig. 3B–E). These findings suggest that PGRN deficiency leads to myelination defects in TDP-43^Q331K/Q331K^ mice. Taken together, PGRN ablation leads to the upregulation of immune pathways and downregulation of myelination pathways in TDP-43^Q331K^ mice, reflecting immune activation and myelination defects caused by PGRN deficiency in TDP-43^Q331K^ mice. Additionally, the axon ensheathment in the central nervous system is also significantly downregulated in TDP-43^Q331K/Q331K^ *Grn*^*−/−*^ mice compared to *Grn*^*−/−*^ (Fig. 3D), indicating that the TDP-43 Q331K mutation might exacerbate myelination defects in the PGRN-deficient background.

### PGRN deficiency causes mild glial activation and behavioral deficits in TDP-43^Q331K^ mice

**Fig. 4:**
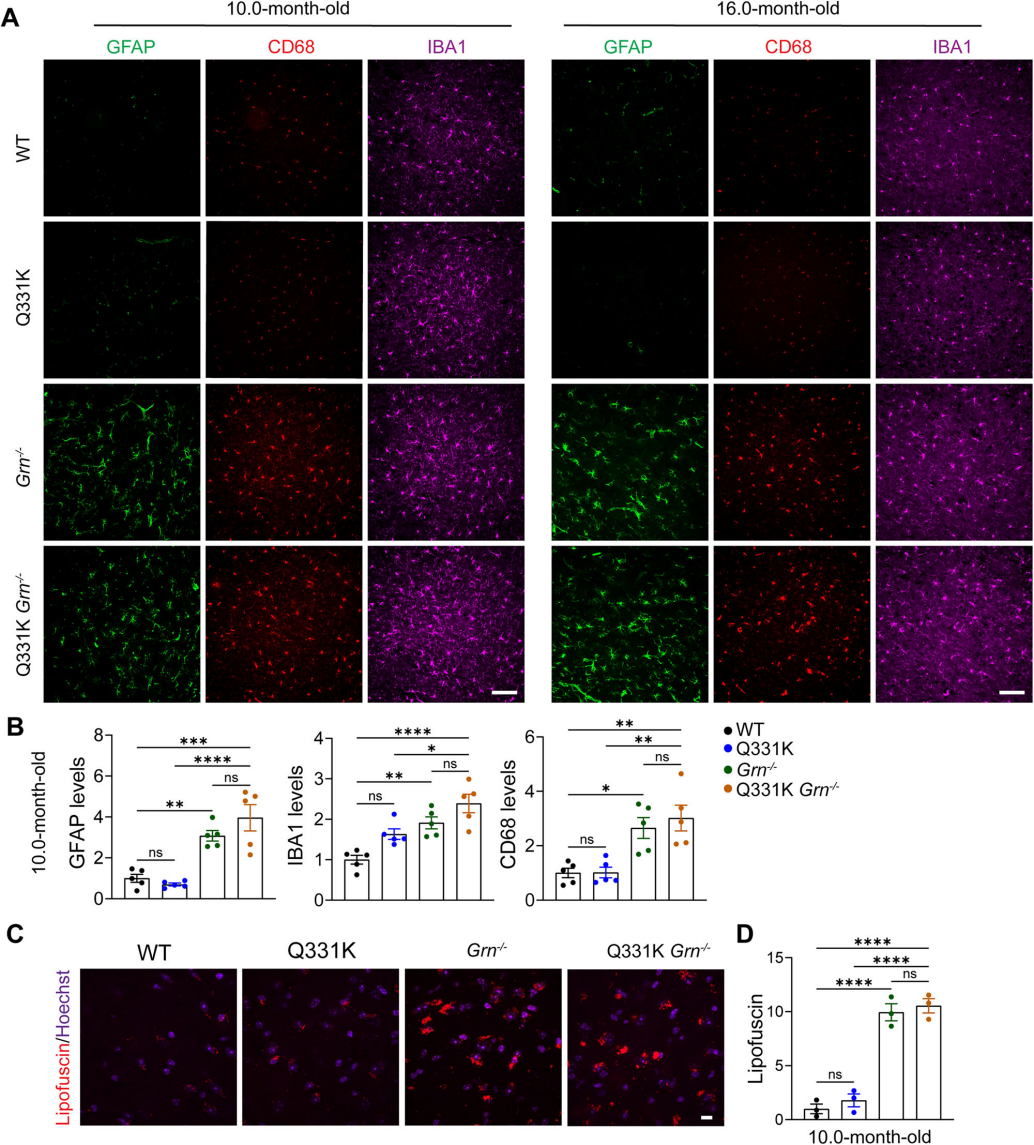
Gliosis and lipofuscin accumulation in TDP-43^Q331K/Q331K^*Grn*^*−/−*^ mice. **A**, **B** Immunostaining of GFAP, CD68, and IBA1 in brain sections from 10 and 16-month-old WT, TDP-43^Q331K/Q331K^ (Q331K), *Grn*^*−/−*^, and TDP-43^Q331K/Q331K^*Grn*^*−/−*^ (Q331K *Grn*^*−/−*^) mice. Representative images from the cortex were shown. Scale bar, 100 µm. Quantification of GFAP, CD68, and IBA1 levels from 10.0-month-old mice sections was shown in (**B**). Data are presented as mean ± SEM from five mice per group (*n* = 5). *p*-values were determined using one-way ANOVA with Bonferroni’s multiple comparisons. *p < 0.05; **p < 0.01; ***p < 0.001;****p < 0.0001. **C**, **D** Lipofuscin accumulation in 10-month-old WT, Q331K, *Grn*^*−/−*^, and Q331K *Grn*^*−/−*^ mice. Representative images for the autofluorescent signals from the thalamus were shown. Scale bar, 10 µm. Quantification of the autofluorescent signals was shown in (**D**). Data are presented as mean ± SEM from three mice per group (*n* = 3). *p*-values were determined using one-way ANOVA with Bonferroni’s multiple comparisons. ****p < 0.0001.

Next, we examined glial activation in TDP-43^Q331K/Q331K^ *Grn*^*−/−*^ mice. A previous study demonstrated microglial activation in the cortex in TDP-43^Q331K^ mouse brain, as evidenced by elevated levels of microglia marker IBA1 and decreased levels of the homeostatic microglia marker TMEM119^34^. While we similarly observed increased IBA1 levels in the cortex of TDP-43 mutant mice, no significant change was detected in the microglial activation marker CD68 (Fig. 4A, B). Consistent with the previous study^34^, we did not observe astrocyte activation in TDP-43^Q331K/Q331K^ mice, as indicated by comparable GFAP levels between mutant and WT mice (Fig. 4A, B). As expected, PGRN deficiency leads to marked glial activation in the mouse brain, as demonstrated by significantly increased levels of IBA1, CD68, and GFAP in the cortex in both 10.0- and 16.0-month-old *Grn*^*−/−*^ mice compared to age-matched WT (Fig. 4A, B). The glial activation was significantly higher in TDP-43^Q331K/Q331K^ *Grn*^*−/−*^ mice compared to TDP-43^Q331K/Q331K^ mice, but only slightly increased relative to *Grn*^*−/−*^ mice (Fig. 4A, B). PGRN deficiency is known to cause lipofuscin accumulation, an indicator for lysosomal dysfunction, in mouse cortex and thalamus^35,36^. To determine whether lysosomal defects are exacerbated in TDP-43 mutant mice under PGRN deficiency, we analyzed autofluorescent signals in the thalamus region of 10-month-old mice. As expected, *Grn*^*−/−*^ mice exhibited significantly increased autofluorescence in the thalamus. However, the signals in TDP-43^Q331K/Q331K^ *Grn*^*−/−*^ mice were comparable to those in *Grn*^*−/−*^ mice (Fig. 4C, D), indicating that TDP-43 Q331K may not exacerbate lysosomal defects caused by PGRN deficiency.

**Fig. 5:**
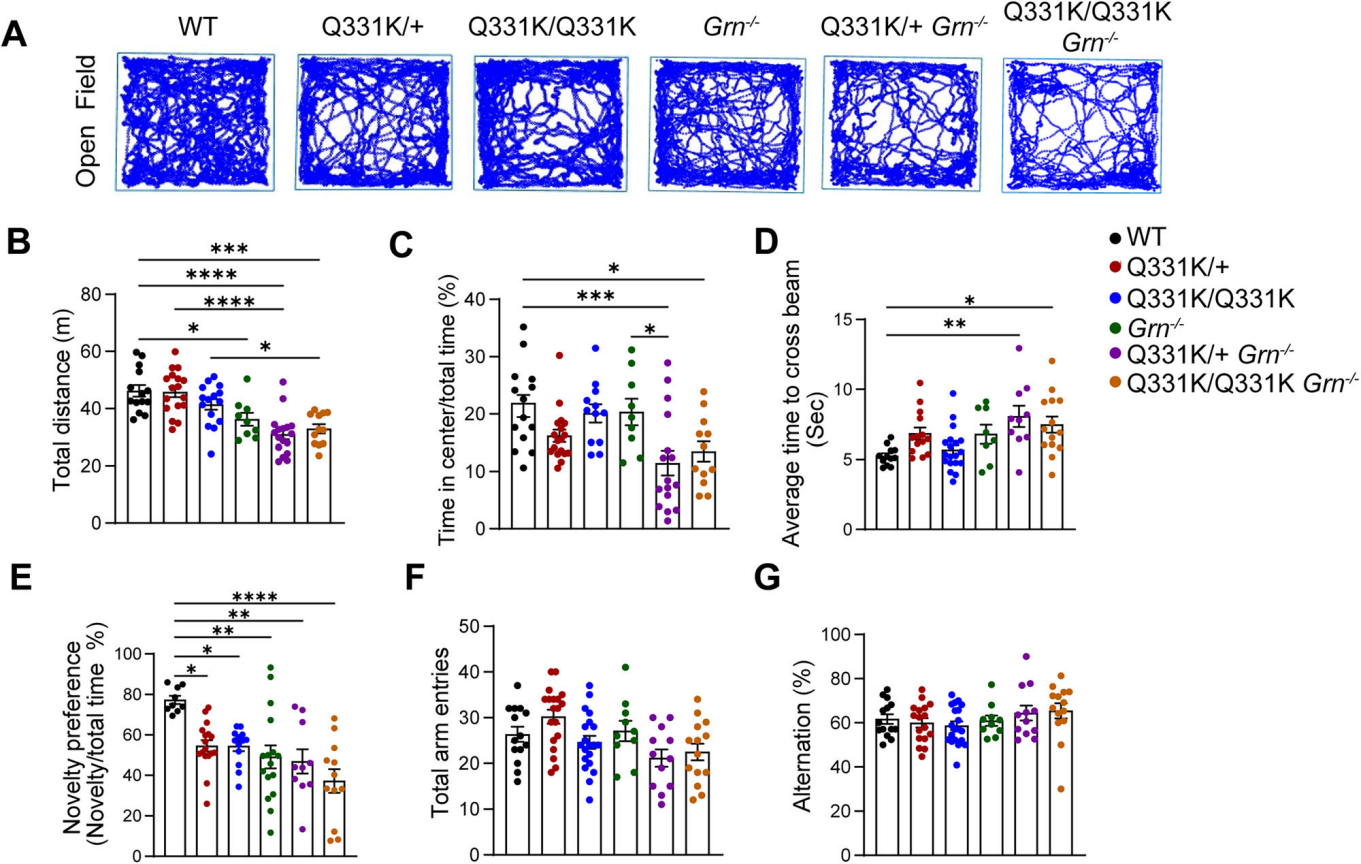
Behavioral deficits in PGRN-deficient TDP-43^Q331K^ mice. **A**–**C** 10-month-old WT, TDP-43^Q331K/+^ (Q331K/ + ), TDP-43^Q331K/Q331K^ (Q331K/Q331K), *Grn*^*−/−*^, TDP-43^Q331K/+^*Grn*^*−/−*^ (Q331K/+ *Grn*^*−/−*^) and TDP-43^Q331K/Q331K^*Grn*^*−/−*^ (Q331K/Q331K *Grn*^*−/−*^) male mice were subjected to the open field test. The total distance the mice traveled (**A**, **B**) and the percentage of the time spent in the center (time in center/total) (**C**) were quantified. Data are presented as means ± SEM (*n* = 9–17). One-way ANOVA tests. **D** Ten-month-old male mice of the indicated genotypes were subjected to balance beam tests. The average time across the beam was shown. Data are presented as means ± SEM (*n* = 8–19). *P*-values were determined using one-way ANOVA tests with Bonferroni’s multiple comparisons and were shown on top of the graphs. **E** 10-month-old male mice with indicated genotypes were subjected to novel object recognition tests. Novelty preference (exploration time of the novel object/exploration time of both objects) × 100%) was shown. Data are presented as means ± SEM (*n* = 9–17). One-way ANOVA tests with Bonferroni’s multiple comparisons. **F**, **G** 10-month-old male mice with indicated genotypes were subjected to Y-maze tests. Total arm entries (g) and alternation (%) (h) were shown. Data are presented as means ± SEM (*n* = 10–20). One-way ANOVA tests with Bonferroni’s multiple comparisons. *p < 0.05; **p < 0.01; ***p < 0.001;****p < 0.0001.

TDP-43^Q331K^ mice display FTLD-like cognitive dysfunction, including executive and memory impairment, but do not develop significant motor impairments^29^. To determine the effect of PGRN on the motor function in TDP-43^Q331K^ mice, we conducted open field tests to assess the locomotor activity of 10.0-month-old TDP-43^Q331K/Q331K^ *Grn*^*−/−*^ and TDP-43^Q331K/+^ *Grn*^*−/−*^ mice. The total distance traveled by both TDP-43^Q331K/Q331K^ and TDP-43^Q331K/+^ mice was similar to that of the WT mice, while PGRN deficiency in these mice led to a significant reduction (Fig. 5A, B), indicating that PGRN ablation impaired locomotor function in TDP-43^Q331K^ mice. Additionally, both TDP-43^Q331K/+^ *Grn*^*−/−*^ and TDP-43^Q331K/Q331K^ *Grn*^*−/−*^ mice spent less time in the center area of the open field compared to WT mice (Fig. 5C), suggesting that these mice may develop anxiety-like behavior. In balance beam tests, TDP-43^Q331K/Q331K^ *Grn*^*−/−*^ and TDP-43^Q331K/+^ *Grn*^*−/−*^ mice spend more time crossing the beam compared with WT mice (Fig. 5D), indicating motor coordination impairments. For cognitive function assessment, novel object recognition tests and Y-maze tests were performed. TDP-43^Q331K/Q331K^ *Grn*^*−/−*^ and TDP-43^Q331K/+^ *Grn*^*−/−*^ mice exhibit a reduced preference for novelty, similar to TDP-43^Q331K/Q331K^, TDP-43^Q331K/+,^ and *Grn*^*−/−*^ mice when compared to WT mice (Fig. 5E). However, TDP-43^Q331K/Q331K^ *Grn*^*−/−*^ and TDP-43^Q331K/+^ *Grn*^*−/−*^ mice do not show any significant changes in alternation in the Y-maze tests (Fig. 5F, G), suggesting that spatial working memory is not influenced in these mice. In summary, PGRN deficiency causes mild glial activation and behavioral deficits in TDP-43^Q331K^ mice.

### PGRN deficiency does not exacerbate TDP-43 pathology in human TDP-43 transgenic mice

**Fig. 6:**
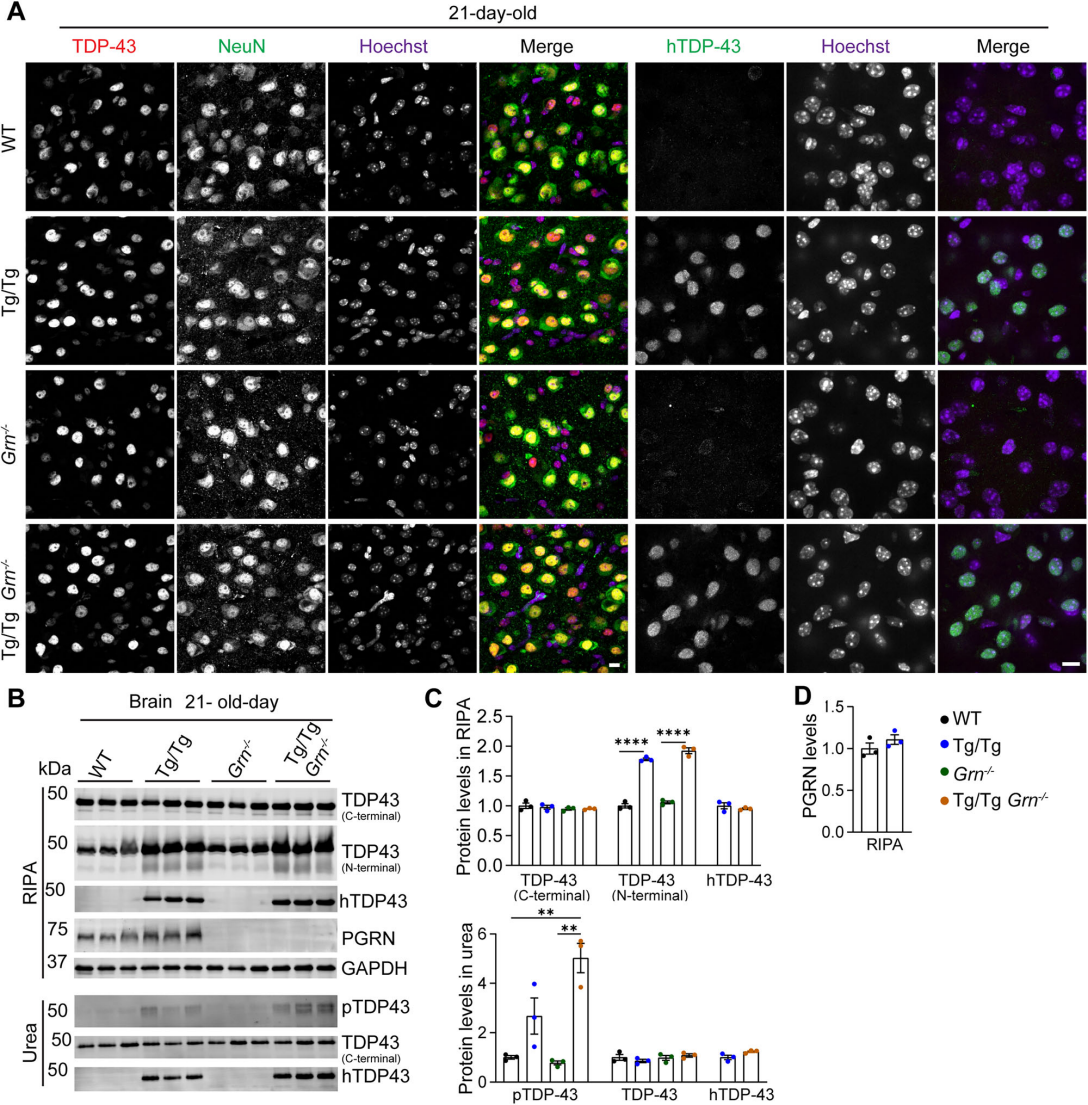
PGRN loss does not exacerbate TDP-43 pathology in 21-day-old hTDP-43^Tg/Tg^ mice. **A** Immunostaining of TDP-43 and NeuN using rabbit anti-TDP-43 CTD antibodies and mouse anti-NeuN antibodies, respectively (left panel) or immunostaining of human TDP-43 (right panel) using mouse anti-human TDP-43 in brain sections from 21-day-old WT, hTDP-43^Tg/Tg^ (Tg/Tg), *Grn*^*−/−*^, and hTDP-43^Tg/Tg^ *Grn*^*−/−*^ mice. Representative images from the cortex were shown. Scale bar, 10 µm. **B** Analysis of TDP-43 and phosphorylated TDP-43 (pS409/410) and PGRN levels in brain lysates from 21-day-old mice. Antibodies recognizing the C-terminal or N-terminal domain of TDP-43 were used to detect total TDP-43 levels. **C** TDP-43 and pTDP-43 levels in RIPA- and urea-soluble fractions were quantified. Data are presented as mean ± SEM from 3 mice per group (*n* = 3). One-way ANOVA tests with Bonferroni’s multiple comparisons. **p < 0.01. **D** PGRN levels in RIPA soluble fractions were quantified and normalized to GAPDH. Data are presented as mean ± SEM from 3 mice per group (*n* = 3).

**Fig. 7:**
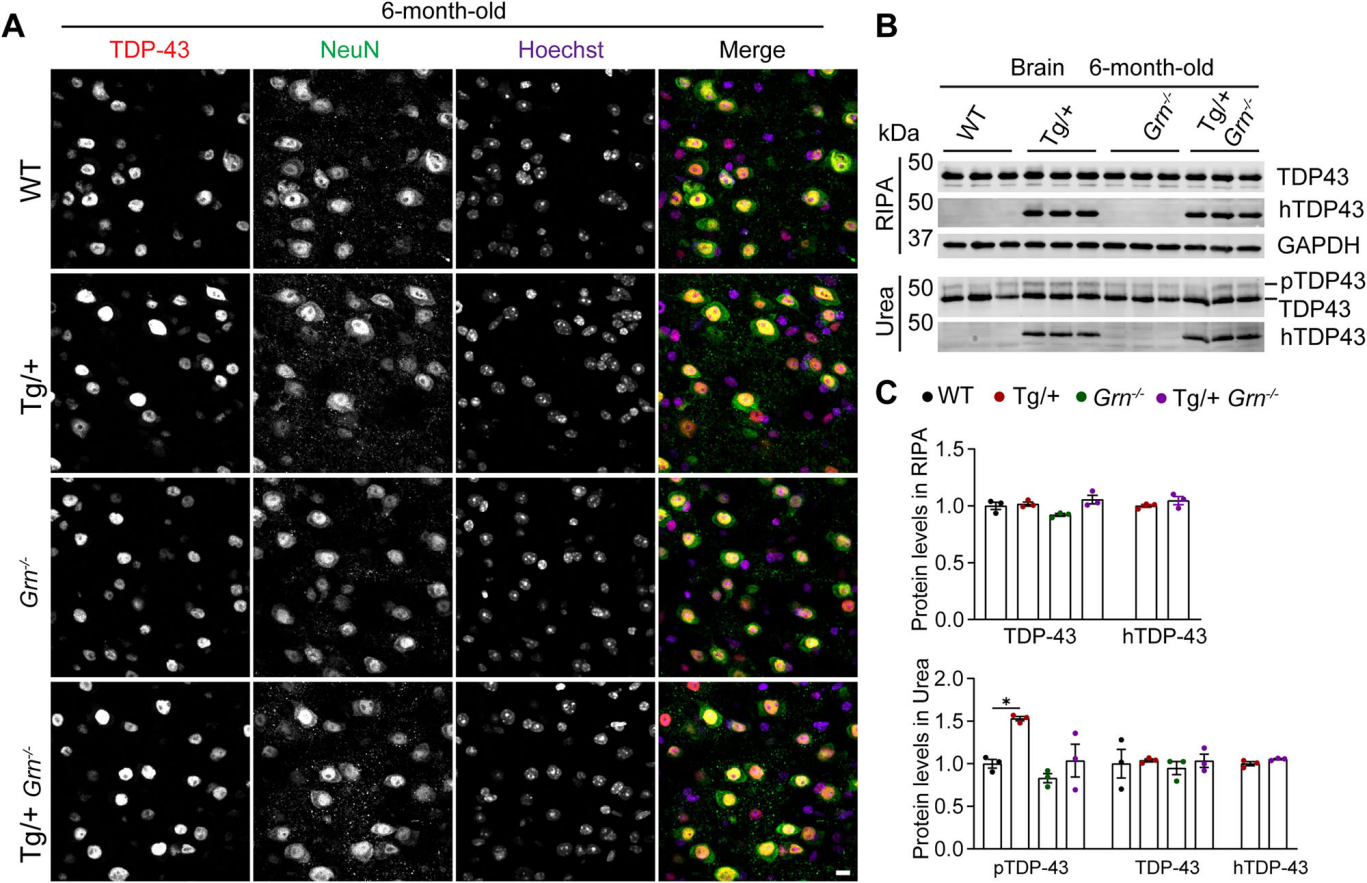
PGRN loss does not exacerbate TDP-43 pathology in 6-month-old hTDP-43^Tg/+^ mice. **A** Immunostaining of TDP-43 and NeuN using rabbit anti-TDP-43 C-terminal domain (CTD) antibodies and mouse anti-NeuN antibodies in brain sections from 6-month-old WT, hTDP-43^Tg/+^(Tg/+), *Grn*^*−/−*^, and hTDP-43^Tg/+^ *Grn*^*−/−*^ mice. Representative images from the cortex were shown. Scale bar, 10 µm. **B** Analysis of TDP-43 and phosphorylated TDP-43 (pS409/410) levels in brain lysates from 6-month-old mice. **C** TDP-43 and pTDP-43 levels in RIPA- and urea-soluble fractions were quantified. Data are presented as mean ± SEM from 3 mice per group (*n* = 3). One-way ANOVA tests with Bonferroni’s multiple comparisons. *p < 0.05.

In addition to the TDP-43^Q331K^ mouse model, we also utilized a mouse model overexpressing human wild-type TDP-43^30^ to determine the effect of PGRN loss on TDP-43 pathology. Heterozygous TDP-43 transgenic mice (hTDP-43^Tg/+^) were crossed with *Grn*^*−/−*^ mice to generate hTDP-43^Tg/+^ *Grn*^*−/−*^ and hTDP-43^Tg/Tg^ *Grn*^*−/−*^ mice. Due to early lethality, hTDP-43^Tg/Tg^ *Grn*^*−/−*^ mice, as well as hTDP-43^Tg/Tg^ mice, were collected at 21 days. hTDP-43^Tg/+^ *Grn*^*−/−*^ mice were collected at 6-month-old. Immunostaining with a rabbit anti-TDP-43 C-terminal domain antibody recognizing both human and mouse TDP-43 and a mouse anti-TDP-43 antibody that specifically recognizes human but not mouse TDP-43 showed no cytoplasmic TDP-43 accumulation in 21-day-old TDP-43^Tg/Tg^ *Grn*^*−/−*^ mice and 6-month-old TDP-43^Tg/+^ *Grn*^*−/−*^ mice (Figs. 6A, 7A), suggesting PGRN loss does not lead to the cytoplasmic mislocalization of TDP-43 in hTDP-43^Tg^ mice. In addition, total TDP-43 levels and human TDP-43 levels, as well as phosphorylated TDP-43 levels in RIPA-soluble and urea-insoluble fractions of the 21-day-old hTDP-43^Tg/Tg^ *Grn*^*−/−*^ and 6-month-old hTDP-43^Tg/+^ *Grn*^*−/−*^mouse brains, were analyzed. A significant increase in phosphorylated TDP-43 (pTDP-43) levels was detected in the urea fraction of 21-day-old TDP-43^Tg/Tg^ and 6-month-old TDP-43^Tg/+^ mouse brains compared to age-matched WT controls, while PGRN loss did not significantly increase pTDP-43 levels in the brain lysates prepared from these mice (Figs. 6B, C, 7B, C). A significant increase in total TDP-43 levels was detected in TDP-43^Tg/Tg^ mice compared to WT mice with the antibody against the N-terminal domain of TDP-43 but not with the antibody against the C-terminal domain of TDP-43 (Fig. 6B, C). This is likely caused by the C-terminal TDP-43 antibody having a higher affinity towards mouse TDP-43 compared to human TDP-43 due to the sequence difference towards the C-terminus of the protein. Nevertheless, loss of PGRN does not alter TDP-43 level or solubility in the 21-day-old hTDP-43^Tg/Tg^ or 6-month-old hTDP-43^Tg/+^ mice (Figs. 6B, C, 7B, C). Additionally, the levels of PGRN in transgenic mice are largely unchanged (Fig. 6B, D). Taken together, our data support that PGRN loss does not exacerbate TDP-43 pathology in human TDP-43 transgenic mice.

### PGRN deficiency does not exacerbate myelin and motor deficits in human TDP-43 transgenic mice

**Fig. 8:**
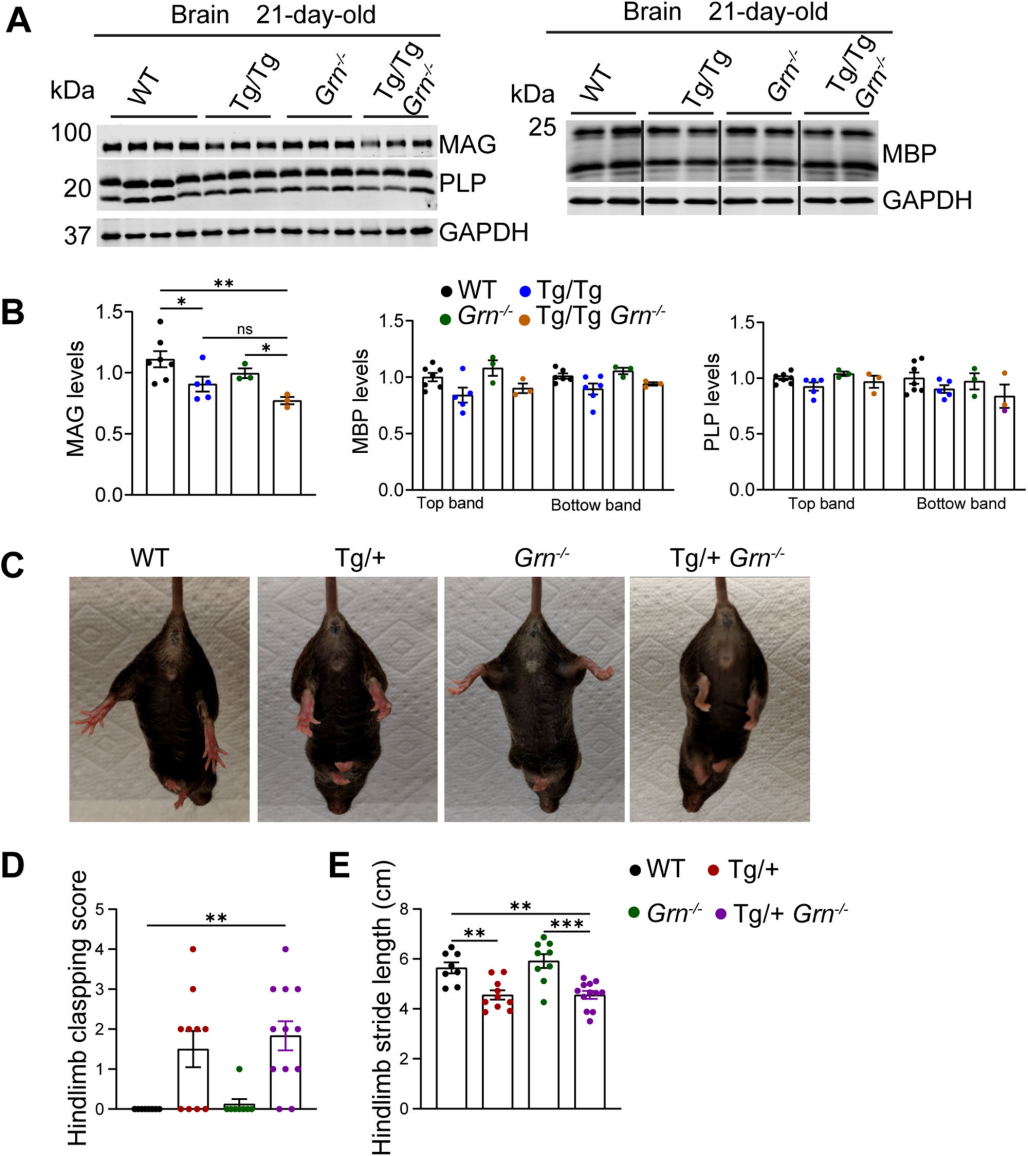
PGRN deficiency does not lead to an obvious alteration in myelination and behavior of hTDP-43^Tg/+^ mice. **A,** **B** Western blot analysis of the myelin markers in the brain. The levels of MAG, MBP, and PLP in 21-day-old WT, hTDP-43^Tg/Tg^, *Grn*^*−/−*,^ and hTDP-43^Tg/Tg^ *Grn*^*−/−*^ mice were analyzed by western blot. The levels were quantified by ImageJ and normalized to the loading control GAPDH. Data are presented as means ± SEM (*n* = 3–7). *p*-values were determined using one-way ANOVA tests with Bonferroni’s multiple comparisons. **C**, **D** The hindlimb clasping test was performed on 6-month-old WT, hTDP-43^Tg/+^(Tg/+), *Grn*^*−/−*^, and hTDP-43^Tg/+^ *Grn*^*−/−*^ (Tg/+ *Grn*^*−/−*^) male and female mice. The hindlimb severity score was quantified. Data are presented as means ± SEM (*n* = 8–12). One-way ANOVA tests. **E** Six-month-old male and female mice were subjected to footprint tests. The hindlimb stride length was measured. Data are presented as means± SEM (*n* = 8–12). *p*-values were determined using one-way ANOVA tests with Bonferroni’s multiple comparisons. *p < 0.05; **p < 0.01; ***p < 0.001.

Our RNA-seq analysis from the TDP-43^Q331K^ mouse model suggested that TDP-43 dysfunction might cause myelination defects. Interestingly, homozygous TDP-43 transgenic mice at the age of 21 days showed decreased levels of myelin proteins, including MAG, MBP, and PLP (Fig. 8A, B). PGRN deficiency does not cause a further decrease in myelin proteins (Fig. 8A, B). The previous study has shown that TDP-43 transgenic mice develop impaired motor function^30^. To determine the effect of PGRN loss on motor function in TDP-43 transgenic mice, we performed hindlimb clasping and footprint tests in 6-month-old mice. We found that hTDP-43^Tg/+^ mice displayed abnormal hindlimb reflexes and reduced hindlimb stride compared to the WT mice (Fig. 8C–E), indicating that these mice develop motor deficits as suggested previously. *Grn*^*−/−*^ mice did not exhibit defects in these tests, while *Grn*^*−/−*^ hTDP-43^Tg/+^ mice showed similar motor impairments as the hTDP-43^Tg/+^ mice (Fig. 8C–E). Taken together, PGRN deficiency does not exacerbate motor deficits in human TDP-43 transgenic mice.

## Discussion

PGRN haploinsufficiency caused by mutations in the *GRN* gene is a leading cause of FTLD with TDP-43 pathology^14–16^. Additionally, the *GRN* gene was identified as a key determinant of LATE, which is characterized by TDP-43 aggregation^17,18^. While the genetic studies point to an important role of PGRN in TDP-43 proteinopathy, it remains to be determined how PGRN regulates TDP-43 proteinopathy.

In mouse models, previous studies have shown that PGRN deficiency alone does not cause any obvious TDP-43 pathology^28,37,38^. Thus, it is worthwhile to investigate whether PGRN deficiency could lead to TDP-43 pathology in mouse models with disrupted TDP-43 protein homeostasis. In this study, we utilized two mouse models of TDP-43, the TDP-43^Q331K^ knock-in and the human TDP-43 transgenic mice, to explore the role of PGRN in TDP-43 proteinopathy. TDP-43 knock-in mouse model harboring the ALS-associated mutation (Q331K) in the endogenous mouse *Tardbp* gene retains the endogenous TDP-43 gene structure^29^. This model does not develop typical TDP-43 pathology but exhibits perturbed TDP-43 homeostasis, which might allow us to investigate whether PGRN loss could induce TDP-43 aggregation in this sensitized background. In addition, TDP-43^Q331K^ mice develop more pronounced FTD-like features^29^, potentially making them a more disease-related model for studying the role of PGRN deficiency in TDP-43 proteinopathy. However, PGRN loss does not seem to affect TDP-43 distribution, total levels and solubility as well as TDP-43’s function in splicing in TDP-43^Q331K^ mouse brain (Fig. 1). In the second model, human TDP-43 transgenic mice, which overexpress wild-type human TDP-43 in neurons, develop TDP-43 aggregates in both the cytoplasm and nucleus of neurons in certain brain regions^30^. By using this model, we aim to determine whether PGRN deficiency could exacerbate TDP-43 pathology. Although phosphorylated TDP-43 was detected in the urea soluble fraction of the 21-day-old hTDP-43^Tg/Tg^ and 6-month-old hTDP-43^Tg/+^ mouse brain lysates via western blot, TDP-43 distribution patterns were similar between transgenic mice and WT mice (Figs. 6, 7). Furthermore, PGRN loss does not significantly impact TDP-43 distribution, levels, or solubility (Figs. 6, 7). Taken together, our findings suggest that PGRN ablation has minimal effects on TDP-43 protein homeostasis in both mouse models used in this study. Future research using improved mouse models will be necessary to better understand how PGRN deficiency might regulate TDP-43 proteinopathy.

Studies have indicated that TDP-43 aggregates can be cleared by autophagy/lysosome pathways^25,39^. It is known that PGRN is essential for proper lysosomal function, and a deficiency in PGRN can lead to lysosomal defects, potentially disrupting TDP-43 protein homeostasis. Previous studies using mouse models and cell cultures have suggested that lysosomal dysfunction caused by PGRN deficiency may contribute to TDP-43 aggregation^40,41^. Additionally, PGRN is highly expressed in microglia, and its deficiency leads to microglial activation, which has been linked to TDP-43 pathology, although the mechanisms remain to be investigated^42^. Furthermore, PGRN-deficient mice also show myelination defects^43^. Myelin debris accumulates in PGRN-deficient microglia, likely due to the impaired lysosomal clearance, which exacerbates lysosomal defects by reducing the levels of lysosomal enzyme cathepsin D and further increases TDP-43 aggregation^40^. These findings support the hypothesis that both lysosomal dysfunction and microglia activation resulting from PGRN deficiency may contribute to TDP-43 proteinopathy. Although PGRN loss did not induce TDP-43 aggregation in TDP-43^Q331K^ mice, our RNA-seq analysis revealed that PGRN deficiency leads to upregulation of immune pathways and downregulation of myelination pathways in the brains of TDP-43^Q331K^ mice (Fig. 3), further supporting a role of PGRN in brain inflammation and myelination. Additionally, previous studies have implicated TDP-43 in myelination. In animal models, perturbation of TDP-43 protein homeostasis by either deletion or overexpression of TDP-43, or induction of cytoplasmic accumulation of truncated TDP-43 fragments in oligodendrocytes, has been shown to cause myelination defects^44–46^. Our findings that the TDP-43 Q331K downregulates myelination pathways in *Grn*^*−/−*^ mice, as well as the observation that the transgenic mice with TDP-43 overexpression in the neurons exhibit reduced levels of myelin proteins in the brain, further suggest a role of TDP-43 in myelination (Figs. 2, 3, 8A, B).

As demonstrated previously, TDP-43^Q331K^ mice display FTLD-like cognitive dysfunction but no significant motor impairment^29^. Consistently, the novel objection recognition test, open field test, and balance beam test suggested that the cognitive function, but not the motor function is impaired in TDP-43^Q331K/+^ and TDP-43^Q331K/Q331K^ mice (Fig. 5). Previous studies have shown that *Grn*^*−/−*^ or *Grn*^*+−/−*^ mouse models develop cognitive dysfunction as well as anxiety or depressive-like behaviors^28,38,47^. Here, we found PGRN deficiency alone leads to both cognitive and motor dysfunction as well as increased open-field anxiety, but these deficits are not significantly worsened in *Grn*^*−/−*^ TDP-43^Q331K^ mice (Fig. 5). Human TDP-43 transgenic mice have been shown to develop motor deficits^30^, as evidenced by abnormal hindlimb reflexes and hindlimb footprints, which was also observed in this study (Fig 8). However, PGRN deficiency does not exacerbate the motor impairment in hTDP-43^Tg^ mice (Fig. 8).

Taken together, our results support that PGRN deficiency or alteration of TDP-43 protein homeostasis leads to behavioral deficits and glial pathology in mice, and PGRN ablation exacerbates these phenotypes in TDP-43^Q331K^ mice. Our results also show a critical role of both PGRN and TDP-43 in regulating the myelination process. However, PGRN deficiency does not have any obvious effect on TDP-43 protein homeostasis seen in human FTLD-*GRN* patients.

## Methods

### Antibodies and reagents

The following antibodies were used in this study: rabbit anti-IBA-1 (Wako, 01919741), goat anti-AIF-1/Iba1 (Novus Biologicals, NB100-1028), rat anti-CD68 (Bio-Rad, MCA1957), mouse anti-GFAP (Cell signaling, 3670S), rabbit anti-TDP43 (Proteintech Group, 12892-1-AP (C-terminal) and 10782-2-AP (N-terminal)), rabbit anti-phospho-TDP-43 (Ser409/410) (Proteintech group, 80007-1-RR), mouse anti-PLP (Millipore, MAB388), mouse anti-MBP (Millipore, SMI-99), rabbit anti-MAG (Proteintech Group, 14386-1-AP), sheep anti-mouse PGRN (R&D systems, AF2557), and mouse anti-GAPDH (Proteintech Group, 60004-1-Ig).

### Mouse strains

*Grn*^*−/−*^ mice^48^ and TDP-43^Q331K^ mice^29^ in the C57BL/6 background and heterozygous transgenic mice overexpressing wild-type human TDP-43^30^ in the C57BL/6/SJL background (TDP-43^Tg/+^) were obtained from the Jackson laboratory. TDP-43^Q331K/+^ or TDP-43^Q331K/Q331K^ *Grn*^*−/−*^ mice were generated by crossing TDP-43^Q331K/Q331K^ and *Grn*^*−/−*^ mice. Human TDP-43^Tg/+^ *Grn*^*−/−*^ or hTDP-43^Tg/Tg^ *Grn*^*−/−*^ mice were generated by crossing hTDP-43^Tg/+^ and *Grn*^*−/−*^ mice. All the mice were housed in the Weill Hall animal facility at Cornell. All animal procedures have been approved by the Institutional Animal Care and Use Committee (IACUC) at Cornell. All applicable international, national, and/or institutional guidelines for the care and use of animals were followed. The work under animal protocol 2017-0056 is approved by the Institutional Animal Care and Use Committee at Cornell University.

### Behavioral test

Ten-month-old WT, TDP-43^Q331K/+^, TDP-43^Q331K/Q331K^, *Grn*^*−/−*^, TDP-43^Q331K/+^*Grn*^*−/−*^ and TDP-43^Q331K/Q331K^*Grn*^*−/−*^ male mice (8–20 mice/group) were subject to the following behavioral tests: (1) Open-field test: Mice were placed in a clean plastic chamber (45 × 45 × 45 cm) for 10 min. The total distance traveled was tracked by the Viewer III software (Biobserve, Bonn, Germany). The apparatus was thoroughly cleaned with 70% ethanol between trials. (2) Balance beam test: Beams that were 80 cm in length were used in the balance beam test for motor coordination. A bright light was used as an aversive stimulus at the start platform, and an escape box (20 × 20 x 20 cm) was placed at the end of the beam. The animals were trained for 3 consecutive trials on each beam for 3 consecutive days, and then were tested on the fourth day. The time taken to traverse the beam was recorded for each trial. The average time to cross the beam of the three trials was calculated. The maximum time cutoff was 60 s. (3) Y-Maze test: Spatial working memory performance was assessed by recording spontaneous alternation behavior in a Y-maze. Y-maze was made of light grey plastic and consisted of 3 arms at 120°. Each arm was 6 cm wide and 36 cm long and had 12.5 cm high walls. Each mouse was placed in the Y maze and allowed to move freely during an 8-min session. The series of arm entries was recorded visually, and arm entry was considered to be completed when the hind paws of the mouse were completely placed in the arm. The maze was cleaned with 70% ethanol after each mouse. Alternation was defined as successive entries into the 3 arms on overlapping triplet sets (e.g., ABC, BCA). The percentage of alternation was calculated as the ratio of actual to possible alternations (defined as the total number of arm entries minus two). (4) Novel object recognition test: Mice were first exposed to two identical objects for 10 min, followed by a 2 h retention interval, and then put back into the same chamber where a novel object was introduced and monitored for 3 min. Exploration, defined as any type of physical contact with an object (whisking, sniffing, rearing on, or touching the object), was recorded and analyzed using the tracing software Viewer III. The preference score (%) for the novel object was calculated as (exploration time of the novel object/exploration time of both objects) × 100%.

Six-month-old WT, hTDP-43^Tg/+^, *Grn*^*−/−*^, TDP-43^Tg/+^*Grn*^*−/−*^ male and female mice (8-12 mice/group) were subject to the following behavioral tests: (1) Hindlimb clasping test: Mice were suspended by the base of the tail, and their behaviors were recorded for 30 s. Hindlimb clasping was rated from 0 to 3 based on severity^49^. (2) Footprint test: The footprint apparatus consists of a runway (60 cm in length) with a dark box at the end. Mice are trained to walk straight to the dark box on the white paper before the trial. The hind paws of the mice are painted blue. Then the mice are made to walk on the white paper. The footprint pattern is analyzed for stride length. Stride length is determined by measuring the distance between each step on the same side of the body. For all behavioral analyses, experimenters were blind to the genotypes of the mice.

### Tissue lysate preparation

Mice were perfused with cold PBS, and tissues were dissected and snap-frozen with liquid nitrogen and kept at −80 °C. On the day of the experiment, frozen tissues were thawed and homogenized on ice with bead homogenizer (Moni International) in ice-cold RIPA buffer (150 mM NaCl, 50 mM Tris-HCl [pH 8.0], 1% Triton X-100, 0.5% sodium deoxycholate, 0.1% SDS) with 1 mM PMSF, and 1x protease inhibitors (Roche). After centrifugation at 14,000 × g for 15 min at 4 °C, supernatants were collected for analysis. The insoluble pellets were washed with RIPA buffer and extracted in 2× v/w of Urea buffer (7 M Urea, 2 M Thiourea, 4% CHAPS, 30 mM Tris, pH 8.5). After sonication, samples were centrifuged at 200,000 g at 24 °C for 1 h, and the supernatant was collected as the urea-soluble fraction.

### Immunofluorescence staining, image acquisition, and analysis

For mouse brain section staining, mice were perfused with cold PBS, and tissues were post-fixed with 4% paraformaldehyde (PFA). After dehydration in 30% sucrose buffer, tissues were embedded in the O.C.T compound. Brain sections with an 18-µm-thickness were cut with a cryotome. Antigen retrieval was performed by microwaving in citrate buffer (pH 6.0) for 15 min. Tissue sections were blocked and permeabilized with 0.1% saponin in Odyssey blocking buffer before incubating with primary antibodies overnight at 4 °C. The next day, sections were washed 4 times with PBS, followed by incubation with secondary fluorescent antibodies and Hoechst at room temperature for 2 h. The sections were treated with the Autofluorescence Quencher (TRUE Black) to quench the autofluorescence. The slides were then mounted using a mounting medium (Vector Laboratories). Images were acquired on a CSU-X spinning disc confocal microscope (Intelligent Imaging Innovations) with an HQ2 CCD camera (Photometrics) using 40x or 63x objectives. Lower magnification images were captured by 10x or 20x objectives on a Leica DMi8 inverted microscope.

For the quantitative analysis of GFAP, CD68, and IBA1 levels in the brain sections, the fluorescence intensity was measured using ImageJ after a threshold application. To quantify the CD68 levels in microglia, IBA1-positive cells were selected, and CD68 signals within microglia were measured using the region of interest (ROI) tool in ImageJ. The mean from the three sections was used to be representative of each mouse. Data from ≥5 brains in each genotype were used for quantification.

### Splicing analysis of the TDP-43 targets using RT-qPCR

Total RNAs were extracted from 10-month-old mouse cortex samples using TRIzol (Invitrogen). One microgram of total RNA was reverse transcribed to cDNA using SuperScript III Reverse Transcriptase (Invitrogen). Quantitative PCR was performed on a LightCyler 480 (Roche Applied Science), and the mRNA levels were measured using efficiency-adjusted ΔΔ-CT. The transcripts analyzed were normalized to β-actin. The primers used to detect the inclusion of *Sort1* exon 17b and *Mapt* exon 2 and 3 were from the previous study^29^. The primer sequences for detecting *Sort1* exon 17b inclusion were: 5′-AACCCCACAAAGCAGGACT-3′ and 5′-CTGCTACGACTGTGACAAGC-3′. The primers for *Mapt* exons 2 and 3 inclusion detection were: 5′-CATGGCTTAAAAGAGTCTCCCC-3′ and 5′-CCTGCTTCTTCGGCTGTAAT-3′ (for exon 2 and 3 included transcripts) and 5′-TTAAAAGCCGAAGAAGCAGGC-3' and 5'-CTGGAGGAGTCTTAGGGCTG-3′ (for exon 2 and 3 skipped transcripts). The primers for *β-actin* were: 5′-ACGAGGCCCAGAGCAAGAG-3′ and 5′-TCTCCAAGTCGTCCCAGTTG-3′.

### RNA-seq analysis

Total RNA was extracted from the brain cortex of 10-month-old mice using Trizol (Thermo Scientific). RNA quality was checked using Nanodrop, gel electrophoresis, and Agilent Fragment Analyzer. RNAseq libraries were generated by the Cornell TREx Facility using the NEBNext Ultra II Directional RNA Library Prep Kit (New England Biolabs) using 700 ng input total RNA per sample. At least 40 M reads (2x150nt PE) were generated on a NovaSeq (Illumina). Reads were trimmed to remove low-quality and adaptor sequences with TrimGalore (a wrapper for cutadapt and fastQC), requiring a minimum trimmed length of 50 nt. Reads that passed quality control were aligned to the reference genome (mouse GRCm38/mm10)^50^ using STAR^51^, using ‘--quantMode GeneCounts’ to output counts per gene. SARTools^52^ and DESeq2^53^ were used to generate normalized counts and perform differential gene expression analysis. Differentially expressed genes (DEGs) were identified by false discovery rates (FDR) or adjusted p-value ≤ 0.05 using multiple testing with Benjamini-Hochberg correction.

Heatmaps of the DEGs were generated by using Heatmapper^54^. An enrichment analysis using the hallmark gene sets or gene ontology biology process (GOBP) was performed using Gene Set Enrichment Analysis (GSEA)^55^. The normalized counts of all the expressed genes were uploaded to GSEA, and a standard GSEA was run with recommended default settings (1000 gene set permutations). The FDR was estimated to control the false-positive finding of a given normalized enrichment score (NES) by comparing the tails of the observed and null distributions derived from 1000 gene set permutations. The gene sets with absolute normalized enrichment score (NES) > 1.0, nominal p-value (NOM p-val) < 0.05, and false discovery rate q value (FDR q-val) <0.1 were considered as significantly enriched. The subset of genes that contributes most to the enrichment result was defined as the core enrichment genes.

### Statistics analysis

Statistical analysis was performed using GraphPad Prism 10. Data are presented as mean ± SEM. Statistical significance was assessed by unpaired two-tailed Student’s t-test (for two-group comparison) and one-way ANOVA tests (for multiple comparisons), followed by Bonferroni’s multiple comparisons post hoc tests. P values less than or equal to 0.05 were considered statistically significant. The details about the sample sizes and number of replicates in each experiment are given in the respective sections of the methods and figure legends. *p < 0.05; **p < 0.01; ***p < 0.001.

## Supplementary information

Supplementary Information

Supplementary Information

## Data Availability

The data supporting the findings of this study are included in the supplemental material. A complete list of genes identified in RNA-seq is provided in Supplementary Data 1. Uncropped western blot images are provided in Supplementary Fig.2. Additional data are available from the corresponding author on request.
