# Supplementary material for: Progranulin deficiency does not exacerbate TDP-43 pathology in TDP-43 transgenic mouse models
Source: NPJ Dement. 2025 Jul 21;1(1):16. doi: 10.1038/s44400-025-00020-4 (PMC12279532; doi:10.1038/s44400-025-00020-4)
Supplement: Supplementary file 1 — Supplementary Information [file 44400_2025_20_MOESM1_ESM.pdf]

Supplementary Information:

Progranulin deficiency does not exacerbate TDP-43 pathology in TDP-43 transgenic mouse models

Cha Yang, Tuancheng Feng and Fenghua Hu[#]

Supplementary Figure 1 and Figure 2.

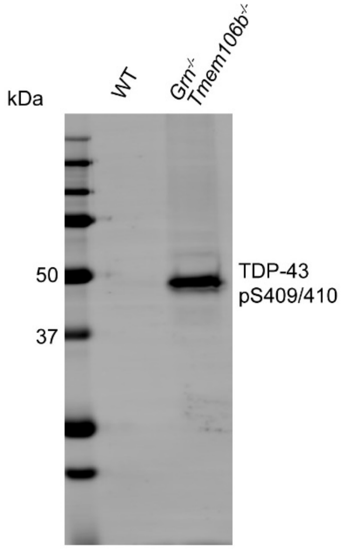

Fig.S1: Validation of the anti-TDP-43 pS409/410 antibody. Phosphorylation levels of TDP-43 in the urea-soluble fraction from the PGRN and TMEM106B double knockout brain samples were measured using a rabbit anti-phospho-TDP-43 antibody (Ser409/410) (Proteintech group, 80007-1-RR).

Fig.S2: Uncropped western blot images for Fig.1B, Fig.6B, Fig.7B and Fig.8A

Fig.1B:

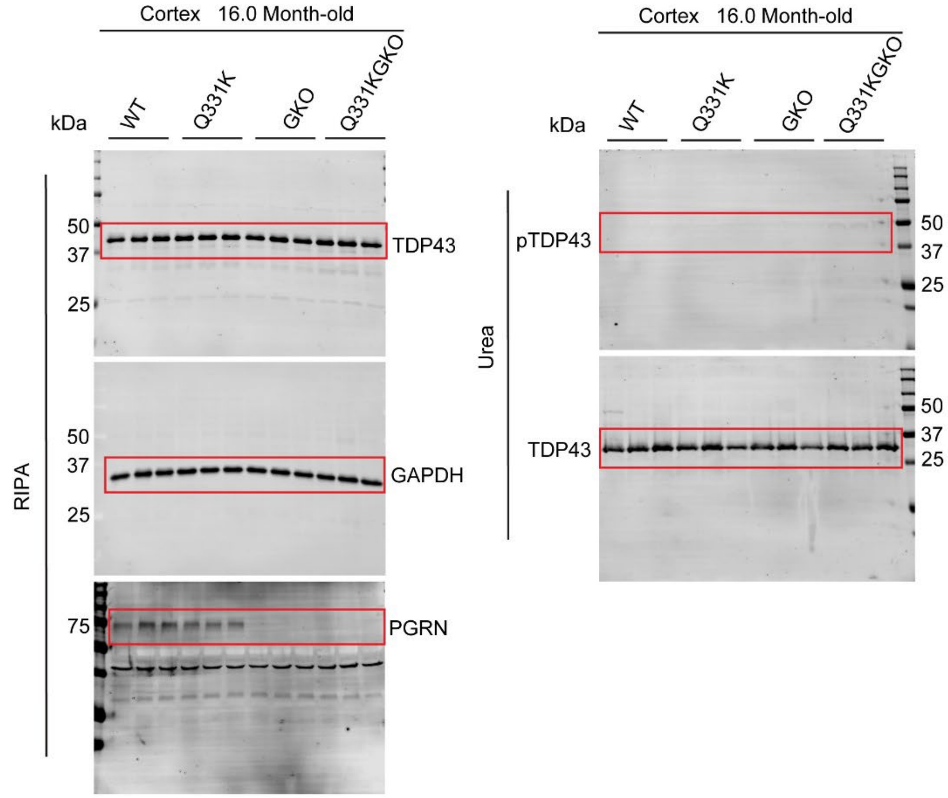

Fig.6B:

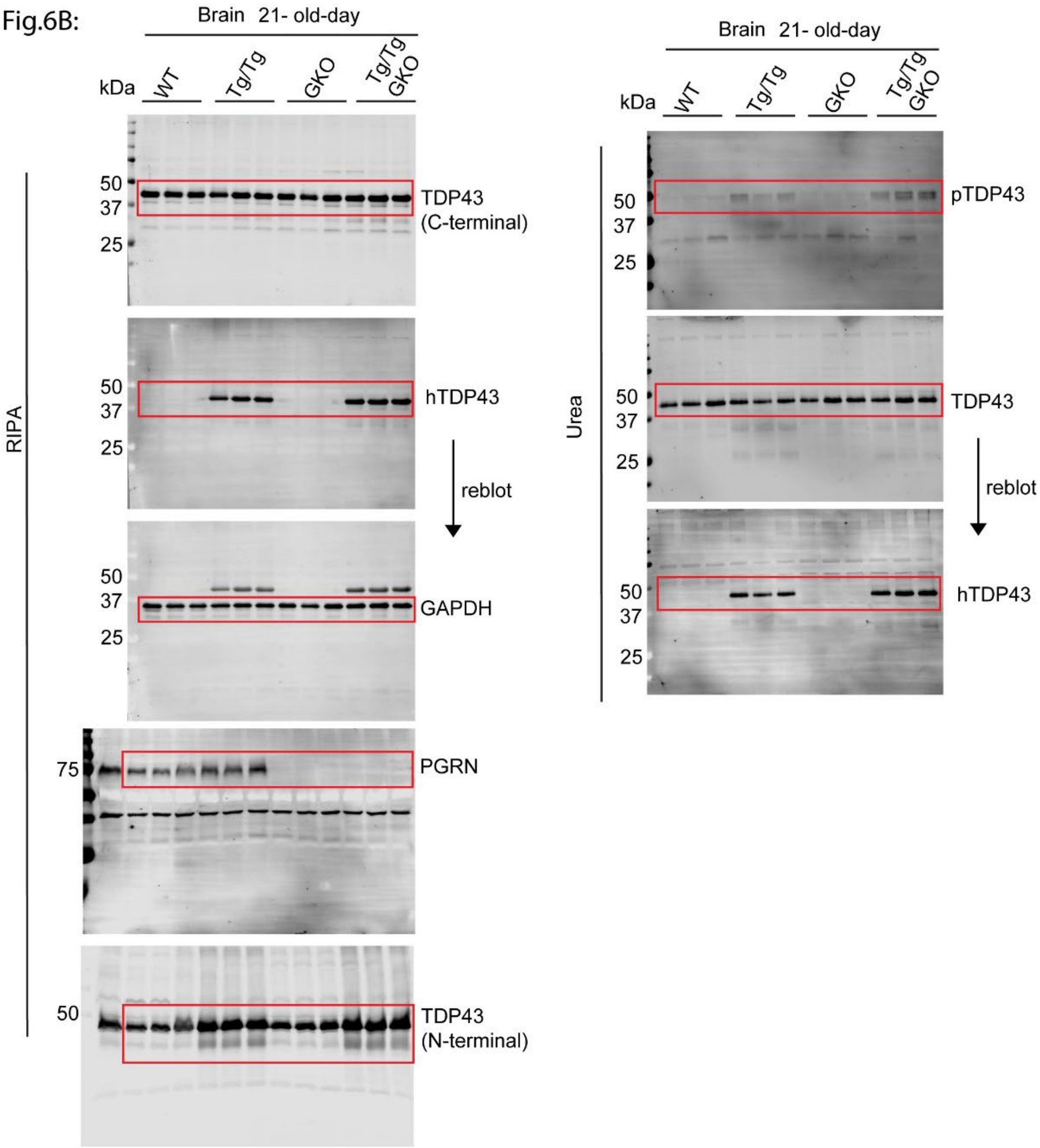
Brain 21- old-day
kDa
WT
Tg/Tg
GKO
Tg/Tg GKO
RIPA
50
37
25
TDP43 (C-terminal)
hTDP43
reblot
GAPDH
75
PGRN
TDP43 (N-terminal)
Urea
pTDP43
TDP43
hTDP43

Fig.7B:

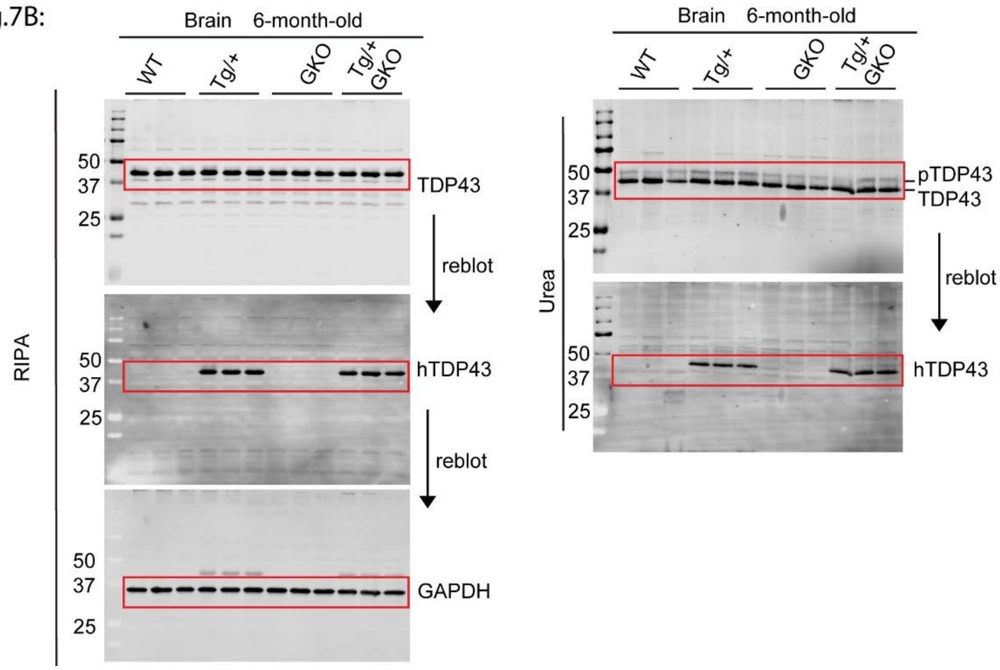
Brain 6-month-old
WT
Tg/+
GKO
Tg/+ GKO
RIPA
50
37
25
TDP43
reblot
hTDP43
reblot
GAPDH
Urea
pTDP43
TDP43
hTDP43

Fig.8A:

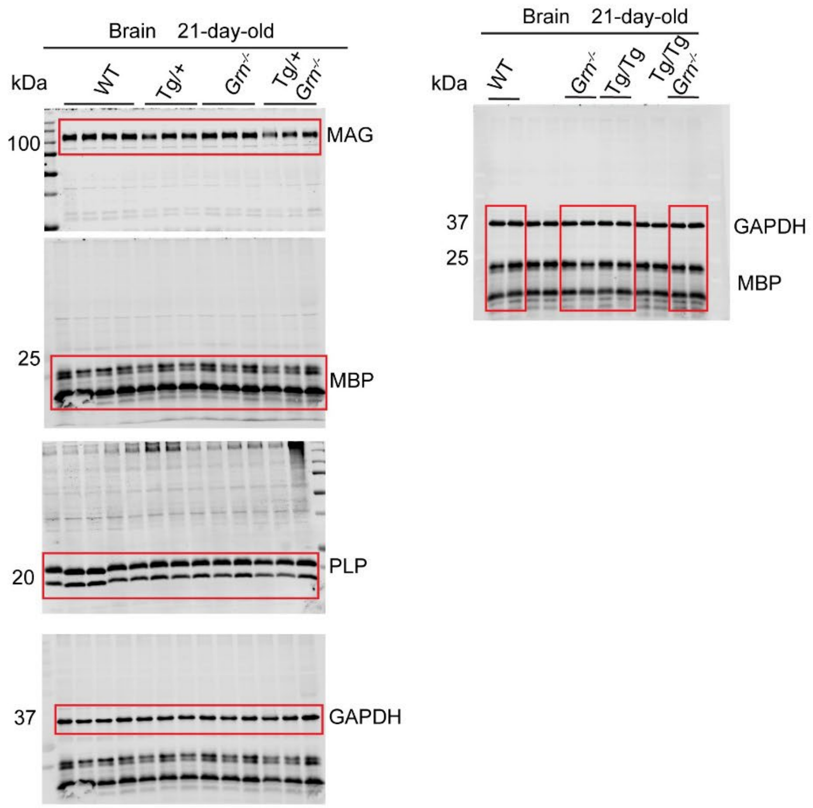
Brain 21-day-old
kDa
WT
Tg/+
Grn-/-
Tg/+ Grn-/-
100
MAG
25
MBP
20
PLP
37
GAPDH
Tg/Tg
Tg/Tg Grn-/-
